# An Autonomous Solar-Powered Marine Robotic Observatory for Permanent Monitoring of Large Areas of Shallow Water

**DOI:** 10.3390/s18103497

**Published:** 2018-10-17

**Authors:** I. González-Reolid, J. Carlos Molina-Molina, A. Guerrero-González, F. J. Ortiz, D. Alonso

**Affiliations:** 1 Department of Automation and Systems Engineering, Universidad Politécnica de Cartagena, Plaza del Hospital 1, 30202 Cartagena, Spain; inocencio.reolid@gmail.com (I.G.-R.); jcarlos.molina@upct.es (J.C.M.-M.); antonio.guerrero@upct.es (A.G.-G.); 2 DSIE Research Group, Universidad Politécnica de Cartagena, Plaza del Hospital 1, 30202 Cartagena, Spain; diego.alonso@upct.es

**Keywords:** ASV, autonomous vehicles, fuzzy controllers, solar vehicle, long term, robotic marine observatory, permanent presence

## Abstract

Apart from their ecological value, the world’s oceans are among the planet’s most valuable resources, a rich source of food and wealth and in urgent need of protection. This article describes BUSCAMOS-RobObs, a robot-based observatory, consisting of an autonomous solar-powered marine robot with specialized sensing systems designed to carry out long-term observation missions in the inland sea of the Mar Menor in southeastern Spain. This highly specialised device is unique because it has the capacity to anchor itself to the seabed and become a “buoy”, either to take measurements at specific points or to recharge its batteries. It thus avoids drifting and possible accidents in the buoy mode, especially near the coast, and resumes monitoring tasks when the required energy levels are reached. The robot is equipped with a broad range of sensors, including side scan sonar, sub-bottom sonar, laser systems, ultrasound sonar, depth meters, a multi-parametric probe and a GPS, which can collect georeferenced oceanic data. Although various types of autonomous vehicles have been described in the literature, they all have limited autonomy (even in the long term) as regards operational time and covering the seabed. The article describes a permanent monitoring mission in the Mar Menor, with a combination of solar energy and a decision-making strategy as regards the optimum route to be followed. The energy and mission simulation results, as well as an account of actual monitoring missions are also included.

## 1. Introduction

The world’s seas are a valuable resource as well as a key element in its ecology, and are in need of protection as an important source of food, wealth and life. For this, systems and technologies are needed to monitor their status and guarantee their sustainable management. The successful management of marine resources involves monitoring the physical and chemical parameters related to water quality, such as salinity, temperature, dissolved oxygen, nitrates, density, and chlorophyll levels, among others. Other reasons for monitoring the seabed include, detecting and preserving artefacts of archaeological value, monitoring the status of marine flora and fauna, especially sensitive species in danger of extinction, and detecting and removing man-made contaminants and plastics. This last point is particularly important as over time plastic fragments into microplastics, which are toxic to both marine life and humans. According in [1], in 2050 there will be more tons of plastic waste than fish in the oceans. The EU has declared war on plastic waste and is preparing a recycling strategy in [2] to ensure that every piece of packaging on the continent is reusable or recyclable by 2030, with an investment of €350 M over the next few years. 

Although oceanographic observatories can monitor large expanses of water continuously and efficiently, and are normally based on static buoys or wireless or wired underwater sensor networks, they can only take measurements at fixed points. When large areas have to be covered specific operations have to be planned and executed. The cost, in terms of time and money, of such operations is normally high and in proportion to the area monitored. The deployment of a large number of buoys is unfeasible due to their impact on the environment and their vulnerability to vandalism. To obtain trustworthy readings and avoid errors when extrapolating measurements to other areas it is fundamental to employ appropriate purpose-built monitoring systems.

Marine robots, like Remotely Operated Vehicles (ROVs), Autonomous Underwater Vehicles (AUVs), or Autonomous Surface Vehicles (ASVs), do not suffer from the main limitations of buoys and are now available for a wide range of observation missions. However, they all have limited autonomy in terms of both time and the area they can cover. In addition, they must be transported to the observation area, deployed for a certain time and picked up when the mission is over, all of which involve extra costs.

The power issue is one of the fundamental challenges of increasing AUV mission times. Renewable energy sources must be used because fuel-based solutions will run out of fuel sooner or later. The current photovoltaic solar systems can generate around 1754 W/m^2^ in good weather conditions. With the limited ASVs recharging surface area, this energy is not enough to keep the thrusters and control systems in permanent operation on long missions and recharging operations must be as efficient as possible to minimise standby times. These operations can be difficult in bad weather conditions, may require more power to avoid drifting than what is effectively injected into the batteries, and may even result in a system shutdown.

From the detailed review of the state of the art in Section 2, we conclude that no solution involving autonomous vehicles has yet been proposed that guarantees a permanent presence in the exploration area. Given the limitations of both types of existing solutions (buoys and autonomous marine vehicles) in this paper we propose a novel approach to explore shallow waters, which we call the BUSCAMOS Robotic Observatory (BUSCAMOS-RobObs). The system is a combination of autonomous vehicles and fixed buoys, whose complete power and navigation autonomy is achieved thanks to a fuzzy decision-making software architecture.

The proposed solution has an ASV’s freedom of movement and can anchor itself to the seabed and become a “buoy”, either for measuring purposes or recharging the batteries, thus ensuring the energy autonomy required for carrying out permanent operations. The vehicle thus avoids drifting and possible accidents in the “buoy mode”, especially important near the coast, and resumes monitoring tasks when the required power levels are reached, all of its operations being managed by the vehicle’s central control and decision-making system. The decision-making system calculates the route to the next area to be explored taking into account a number of parameters, including: vehicle position, the water’s physical and chemical parameters, distance to the next exploration area, solar radiation, available energy, wind speed and direction, water currents, etc.

This article describes the hardware and software innovations included in BUSCAMOS-RobObs. To demonstrate the feasibility of the proposed solution, we include the crucial preliminary simulations of power and decision-making autonomy, together with descriptions of actual observation missions and their results in the Mar Menor (see Figure 6 in Section 4) in southeast Spain. This article is organized as follows: Section 2 outlines the current state of the art and summarises the vehicle’s technical specifications, which is described in greater detail in Section 3 (hardware and software, power generation and energy management). The design of the strategic decision-making layer and the application to the observation mission is outlined in Section 4. Section 5 gives details of the tests and their results, while the main conclusions are shown in Section 6.

## 2. State of the Art

This section describes the state of the art of existing technologies that led us to propose a dual approach (buoy-ASV) to a mobile robotic observatory. The two main types of solutions that already exist to carry out marine monitoring are analysed: static systems (sensor networks and buoys) and mobile systems based on autonomous vehicles. Given the aim of designing a vehicle capable of permanent presence in a marine environment, the present state of the art of the methods proposed to improve the energy autonomy of autonomous systems is given special attention.

### 2.1. Static Monitoring Solutions: Buoys and Sensor Networks

According to [3], oceanographic observatories measure physical, chemical, and biological variables in coastal waters, perform real-time observations and supply information to help in decision-making on managing problems such as climate change, the human impact, coastal variability, natural disasters, or the condition of ecosystems. They normally consist of fixed buoys with different sensors to measure the parameters of interest. The present authors advocate using Wireless Sensor Networks (WSN) as an innovative solution for the deployment of multiple buoys in coastal areas, as they are cost-effective and have low energy consumption (powered by solar panels), which allows a higher number of sensors to be deployed with different network topologies and protocols at a reasonable cost in shallow coastal waters.

The review of wireless sensor networks in marine environment monitoring described in [4] makes interesting comparisons of the leading current marine wireless sensor networks and conclude that when a buoy-based system is used for long-term observation the sensors are susceptible to bio-fouling (microbial and algal films). Other problems highlighted in these articles include the need for flotation and mooring devices, possible vandalism, interference from maritime traffic, the movement of nodes by tides, waves, vessels, etc., and the fact that an anchor is required to fix them to the seabed. Energy systems for sensor nodes generally include batteries, capacitors, thermal engines, fuel cells and solar energy collectors. The photovoltaic system is the most frequently used energy system at present in [5]. However, using batteries to store energy has many limitations, such as a restricted energy capacity, the low energy levels obtained from solar panels or excessive consumption in [6]. When the buoys run out of energy they stop transmitting. Exhausted batteries made of toxic heavy metals are a highly contaminating waste. Rechargeable batteries with an energy capacity greater than the daily energy consumed by the system are a better option and enable the power system to supply energy at night and in bad weather conditions in [7].

Most of the aforementioned papers provide little information on power systems in general, and the energy management algorithms, if they exist, are limited to considering battery voltage and intensity and maybe the power obtained from the solar panels. In most cases small panels (2.5 W/8 V) are installed with 3.7 V/5 Ah batteries, as in [4]. Some authors monitored the energy consumption of their electronic and electrical equipment in [8], but did not use the information to create an intelligent control system for the diverse power requirements.

It should be noted that all these systems have a serious limitation: a deployed buoy can only provide values from its fixed measuring point. A number of buoys will be needed to cover a wider area with costs rising in proportion to the area monitored, unfeasible due to the impact on maritime traffic, the environment, and tourism.

### 2.2. Autonomous Monitoring Solutions: Marine and Underwater Vehicles

Due to the intrinsic complexity of the marine environment, monitoring tasks in the open sea started with remotely operated vehicle (ROVs) and continued with the development of autonomous surface and underwater vehicles (ASVs and AUVs), which overcome the ROVs’ main limitation: the need for a support vessel and a human controller. AUVs thus opened up new opportunities for sea monitoring and explorations as they could gather data autonomously for extended periods of time at a lower cost than ROVs.

To make the most of AUVs they must be able to carry out long missions, an important goal of research in autonomous systems in general, especially in marine robots (see for example the Special Issue on “Long-Term Autonomy in Marine Robotics” in [9]). According to [10], long-term autonomy can be defined as “the ability of a robot to operate robustly for an extended period of time (hours, days or even weeks), with reduced human supervision and in a real environment”. Long-term autonomy can be considered from three points of view [11], all related to the problems of operating robots over long periods of time: (i) changing state of the sea and the perishability of current knowledge; (ii) unforeseen hardware and software failures during extended operations; and (iii) unexpected changes in mission goals, or new constraints and priorities for the robot to execute, which can also cause conflicts.

Achieving any of the aforementioned levels of autonomy requires first of all achieving the maximum possible power autonomy. This section describes the main initiatives in the literature to develop marine observation tasks, with special emphasis on the energy management system used for long-term operations at sea.

Gliders are, perhaps, the best example of marine vehicles in terms of energy autonomy. They use hardly any energy to move and by moving their wings and modifying their buoyancy they can convert vertical immersion movements into a horizontal movement, thus obtaining a very low power consumption. Although they are not as fast as other AUVs, gliders have a greater time and distance range than propeller-powered vehicles. Their measurement missions can last for months and cover thousands of kilometres. The so-called Wave Gliders in [12] use the force of the waves to advance and do not use any thrusters. They consist of two parts joined by a cable: a surfboard floating on the surface and a second part about 8 m under the water. This system also uses solar panels to power the on-board electronics.

In general, Glider-type solutions are not suitable for exhaustive explorations of the marine environment, as these vehicles are at the mercy of currents and waves. Since they require little power to operate they have less accuracy regarding the area to be monitored. A system described in [13] proposes a solution to this problem: an optimal mission-planning algorithm for a fleet of gliders based on the sampling on-demand paradigm. In this strategy, the user quantitatively sets the requirements related to the uncertainty over an area of interest that needs to be achieved by means of measurements taken by observing assets. In general, the uncertainty can vary from one region to another in the study area, according to the scientific/operational requirements of the mission. For instance, some areas may be considered more important to explore than others.

The more precise solutions, which provide better control of the areas explored, require the use of thrusters, and thrusters require a complete energy management strategy to ensure long periods of AUV activity. The remainder of this section summarises some of the AUVs described in the literature in order of decreasing autonomy. For each vehicle, we describe, when possible, its purpose, sensors and on-board electronics, measured mission time, energy source/s available and their management, and whether the system software takes into account energy issues when planning the missions.

SAUV II in [14] is an exclusively solar-powered AUV, relatively small in size, with a total solar panel surface of 1 m^2^. The vehicle has a lithium battery (32 V, 2 kWh) as its energy storage system, which stores energy in the daytime to execute the missions at night. SAUV was designed to carry out long-endurance missions, such as monitoring, surveillance, or station-keeping. The longest mission recorded lasted for 30 days with 8 h of continuous operations. 

A catamaran for environmental exploration in Rhode Island is described in [15]. Given its small size, it can venture close to the shore. It has a few sensors to capture the physical-chemical parameters of the water and carries a radar sensor, GPS, altimeter for detecting the seabed and is controlled by two CPU based on Linux and a Windows operating system. The energy system is composed of a 5.5 kW diesel generator, which accumulates the energy in 4 batteries 12 V, which provide the energy to 48 V electric motors. Navigation time is limited to about 21 days.

The USV described in [16] was designed and built to perform extended missions for environmental research by using an efficient route planner with Voronoi diagrams and a Dijkstra search algorithm. However, this algorithm does not take into account the solar energy generated to plan the most efficient route. The vessel is equipped with a GPS, AIS transponder, radar reflectors, depth sounder and navigation lights. Maximum navigation time is 3 months, powered by 1200 W solar panels, a wind turbine system with a power output of 720 W, and a 2.5 kW diesel generator system to recharge the batteries.

The catamaran described in reference [17] has two electric motors powered by a mere 300 Wp solar module. Maximum navigation time is about 24 h. The ASV is equipped with a set of navigation sensors that includes a GPS, compass, depth sensor, laser scanner, camera, optical methane detector (OMD), a YSI Sonde (measures temperature, conductivity, chlorophyll, turbidity, dissolved oxygen, incident radiation), wind sensor and a profiled sonar, and laser-based obstacle-avoidance sensor. A profiling arm allows measurements of up to 5.5 m while the vehicle is moving. 

Another vehicle used for shallow water and bathymetric studies includes a purpose-built software with integrated navigation and data acquisition systems in [18]. This autonomous vessel has a navigation time of only two to four hours. As sensors, it carries a GPS, surface and submerged camera for video acquisition, ultrasonic obstacle detection, temperature control system, single beam echo sounder, inertial platform, an OLinuXino microcomputer, with a Linux operating system.

The vehicle described in [12] also has a reduced autonomy (up to eight hours) although it is equipped with high-density rechargeable batteries. It carries sensors such as GPS, special electrodes for measuring anomalies in the distribution of electrical resistance along a channel, and temperature and oxygen sensors. According to the authors, it can be used to detect and scare birds away from drinkable water basins and fish-breeding ponds. The control system is based on a Windows OS.

The present authors gained previous experience in developing autonomous marine vehicles. In [19] we describe a multi-vehicle system based on an ASV-UUV combination for oil-spill monitoring, “BUSCAMOS-OIL”. One of its outstanding features is the incorporation of new control strategies based on bio-inspired neural networks to give adaptability and robustness to the ASV and UUV. The two vehicles are connected by an umbilical cable, which allows them to share power and computing resources. With its two robots, the platform has the ability to monitor large tracts of sea both on the surface and in the water column. With its time-series measurements, the system draws precise maps of the oil plume, with information on spill location, size, extent, direction and speed. To ensure its power supply, the ASV contains six photovoltaic panels of 130 Wp each, which recharge two 28 V batteries. It also has a diesel generator when no solar energy is available. This platform has now been improved for carrying out the missions described here, which require greater autonomy. The diesel generator has been eliminated, two more solar panels have been added and the explorer submarine has been removed, thus reducing energy consumption, weight and water friction. The vessel now generates more solar energy, which allows permanent navigation and exploration missions.

Besides stand-alone AUVs and ASVs, there are also other approaches that employ fleets of AUVs/ASVs to monitor and explore the seas. Each vehicle individually manages its energy sources, but as a fleet of vehicles these solutions provide extended capabilities to carry out the aforementioned missions. A fleet of ASVs for assessing greenhouse gas emissions from underwater plants in inland reservoirs, rivers and marshes is described in [20]. Each ASV has certain energy limitations: it uses two 40 W solar modules, stores its energy in 12 V, 20 Ah lithium iron phosphate (LFP) batteries. Although energy management algorithms are not specifically considered in this article, it uses a navigation algorithm in which all the ASVs communicate with each other. At the beginning of the mission they are sent to different locations to minimise power consumption and travelling time.

### 2.3. Dual System: ASV-Buoy

Taking into account the aforementioned problems, two options are therefore available for long-term missions: (i) deploying a large number of fixed marine observatories (buoys) over the area to be monitored (unfeasible due to the impact on maritime traffic, the environment, tourism and cost); or (ii) autonomous systems able to perform permanent monitoring missions with the minimum cost in temporary deployment of vehicles and teams and maximum observation time.

This article describes the solution we have devised for permanent monitoring of lakes or shallow coastal areas, such as the Mar Menor in southeastern Spain (for a description of which see Section 4.1). The system combines the main advantages of autonomous systems and buoys to overcome the above-described limitations and challenges. It can thus be described as a “dual solution robot-buoy”: an autonomous solar-powered marine robotic observatory equipped for data collection during missions, navigation to inspection points, etc. that also has the ability to anchor and become a “buoy”, for either taking measurements at a specific point or prioritizing energy recharge as required, thus avoiding being left to drift with the consequences that it entails (especially near the coast) and returning to monitoring work when the required energy levels are reached. This novel dual robot-buoy configuration ensures a permanent presence in the observation area, as described below. As far as we know, no other similar solutions have yet been proposed.

This robot could also use the Predictive Control Model (PCM) developed by the authors in [21] to obtain optimal battery charging and power consumption, using an algorithm that inhibits fluctuations in the power output of the photovoltaic system due to temperature changes, cloud cover, irradiation, etc. The sinusoidal rotational vector method [22] could also be used to estimate the vessel’s attitude; this method was installed in a land vehicle to determine the difference between the vehicle’s measured and expected attitude.

## 3. General Description of the Dual Observatory

This section briefly describes the fundamental characteristics of the dual robotic observatory BUSCAMOS-RobObs. The following sub-sections will describe in greater detail its energy system, decision-support system, and the hardware and software architecture. The dual system can be defined as a dual autonomous oceanographic observatory, in which *dual* refers to its being either an autonomous vehicle or an oceanographic observatory, as required.

**Figure 1:**
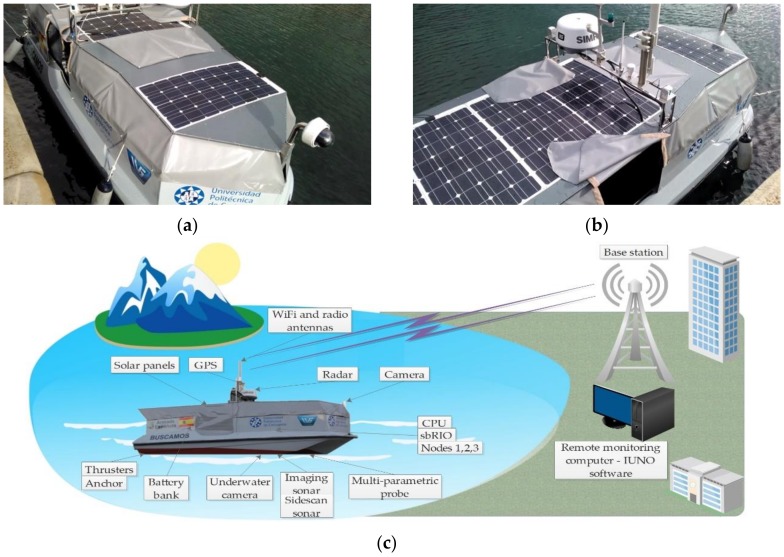
(**a**,**b**) BUSCAMOS-RobObs pictures and (**c**) general scheme.

BUSCAMOS-RobObs is a mono-hull vessel built of polyester reinforced with fiberglass (see Figure 1). The hull is 5.10 m long and 1.97 m wide. The displacement of the vessel under normal operating conditions is approximately 1000 kg, with an average draft of 0.325 m. The vessel is steered by two independent outboard propellers anchored to the transom, where each thruster is powered in series by two 28 V serial connected lithium ion batteries. It is also equipped with a rudder control, which changes the thrust direction of the propellers by means of a linear motor.

The BUSCAMOS-RobObs ASV includes the following sensing devices: a SIMRAD broadband 3G radar, an aft camera (model PTZ AXIS P5534-E), a bow camera (model AXIS P1435-LE), a sidescan sonar (model Tritech SeaKing), an imaging sonar (model Tritech Micron Sonar) and a multi-parametric probe (model YSI 6600 V2). The side scan sonar generates the images of the seabed that are shown in Section 5 (Figure 15), while the imaging sonar and the 3G radar are used for underwater and surface obstacle avoidance. The multi-parametric probe, whose main characteristics are described in [23], can measure, temperature, salinity, density, dissolved oxygen, chlorophyll concentration and nitrates.

The vessel employs a photovoltaic system to produce energy and a set of batteries for the long-term operation of the system. Eight lightweight flexible photovoltaic panels cover an area of 4000 × 1355 mm on the ASV’s deck, with a total installed photovoltaic power of 1040 Wp. As can be seen in Figure 1, the panels’ position on the hull has little effect on the vessel’s aerodynamics and manoeuvrability. Section 3.2 gives further details of the electric system.

The BUSCAMOS-RobObs control hardware architecture is described in Section 3.3, including a detailed description of the main software architectural layers, their roles and responsibilities, while Section 3.4 describes the ASV’s on-board hardware and Section 3.5 the central command, control, communication and computing station. The ASV hardware is composed of three main nodes, interconnected via CAN bus and Ethernet, and with the base station via WiFi, radio and GPRS. The modules are (see Figure 3) as follows:

- *Operational Module*, responsible for the navigation, consisting of a National Instrument sbRIO 9606 main controller and two microcontroller-based electronic cards.
- *Energy Sensing and Management Module*, consisting of an electronic card based on a micro-controller, which acquires information from the different sensors, checking their status periodically and managing energy. It also controls the anchor deployment and retrieval motor.
- *Tactical and Strategic Module*, responsible for operating the decision-making algorithm and storage and management of the acquired data. This module is a single-board DFRobot Lattepanda computer based on the Intel Atom Cherry Trail.

The Energy Sensing and Management Module manages mode changes between autonomous vehicle, fixed oceanographic observatory, or prioritising battery recharging. As described above, one of the dual observatory’s special characteristics is that during battery-charging the vessel is anchored to the seabed with minimum power consumption. When the batteries are fully charged the control system wakes up and starts the scanning process until the batteries return to minimum, when it deactivates the systems. The vehicle thus combines the advantages of buoys and ASVs, while it can also adopt the at-rest mode in bad weather or sea conditions.

### 3.1. Energy Management System

The photovoltaic system configuration is a typical standalone installation. The ASV uses a photovoltaic generator system with eight inboard flexible photovoltaic solar modules of 130 Wp each. With the propellers in power mode the vessel’s systems consume around 1802 Wh. As the photovoltaic solar modules are laid horizontally on the deck, their performance is reduced and they provide around 1040 Wh, insufficient to drive the boat directly from the photovoltaic generator, so that an additional intermediate energy storage system is required. The system stores energy in four batteries 26 V of 104 Amps/unit, distributed in two in-series batteries for each torch propeller, giving 6 h of navigation autonomy, according to Equation (1):

(1) $$\text{Autonomy}=\frac{10816\;\mathrm{Wh}}{1802\;\mathrm{Wh}}=6\;h$$

The battery bank provides stable current and voltage by eliminating transients and provides surge currents to the thrusters when required. Two solar charge controllers are placed between the PVs and the battery bank. These devices adjust the charge rates to the status of the battery bank, applying maximum power point tracker (MPPT) technology. This device boosts the PV lower voltage to charge higher voltage lithium batteries up to 54 V nominal. The characteristics of the photovoltaic system are shown in Table 1.

### 3.2. Decision-Support System

**Figure 2:**
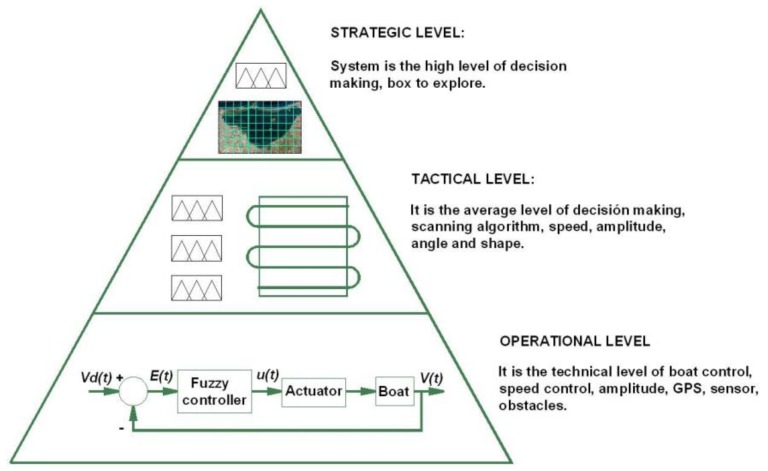
Hierarchical structure of the control system, showing the three levels of control: strategic, tactical and operational.

The control strategy implemented in the ASV is structured into three software layers, as shown in Figure 2:

*Strategic Level*: is the high-level decision-making subsystem for selecting the best geographical area (the “box”) for exploration, according to an analysis of the experimental physical and chemical parameters measured, time elapsed since the previous exploration, energy constraints, and the distance between the present position and the next targeted exploration area. This layer is described in more detail in the following section.

Tactical Level: is the intermediate decision-making system, which receives the next “box” to be explored from the layer above and designs a plan to reach and explore it. It comprises a fuzzy level subsystem to optimize route, speed, course and starting angle. This algorithm processes information from the vessel sensors and the status of the vehicle itself and its environment, such as the tag of the next box to be explored, power available in the batteries, and energy supplied in real time by the on-board photovoltaic system. The GPS coordinates of the next waypoint and thruster speed are sent to the operational level layer, which is the lowest level in the hierarchy.

*Operational Level*: is a low-level purely technical decision-making step focused on navigation control. The system controls the course, speed, angle, etc. and operates the obstacle avoidance sensors. The operational control level uses a fuzzy logic feedback control loop: the data supplied by the tactical level are read and compared with the reference signal, and any errors are used as a control parameter to correct the course.

These decision levels are implemented in a hardware architecture that maintains an equivalent hierarchical order, as described below.

### 3.3. Control Hardware Architecture

**Figure 3:**
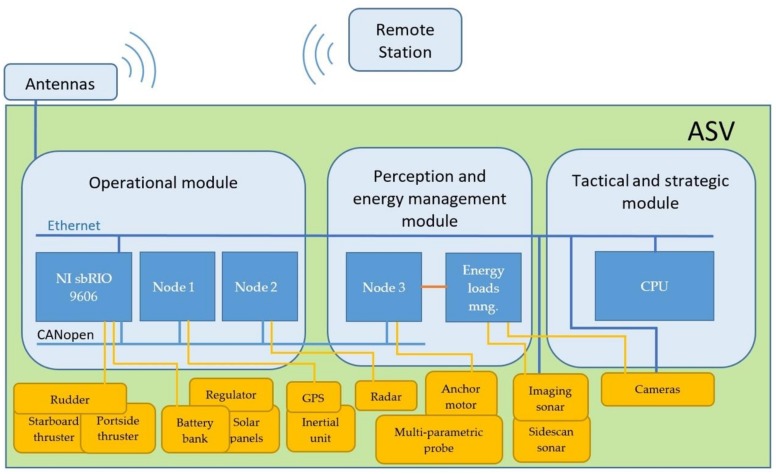
BUSCAMOS-RobObs hardware components and communication networks.

The control hardware architecture consists mainly of three modules, each with a series of electronic devices and cards for specific functions. All the modules are interconnected via a CANopen fieldbus and an Ethernet network (see Figure 3).

The Operational Module is primarily responsible for vehicle navigation: motor control, GPS acquisition, inertial unit heading acquisition, obstacle detection and safety. It consists of a National Instruments sbRIO 9606 main controller and two specifically designed electronic cards, Nodes 1 and 2 of the CANBUS fieldbus, based on a PIC 18f4685-E/PT microcontroller (8 bits and clock frequency of 25 MHz). Each node electronic system includes modules that guarantee the desired functionality (CAN-TTL transducer, serial communication transducer, precision AD converters, voltage regulators, protections, drivers for motor management, etc.).

The Energy Sensing and Management Module (i) acquires information from the multi-parametric probe and cyclically checks its status (operational, maintenance required, etc.); (ii) controls camera and sonar power and (iii) controls the anchor deployment motor, changes vehicle mode between autonomous vehicle, fixed oceanographic observatory, or prioritization of energy recharging. It consists of two custom-designed electronic cards, one of which is the third node of the CANopen fieldbus, and the other an expansion of the previous one, performing load management functions for the different connected devices. The third node has a hardware structure similar to the other two.

The Tactical and Strategic Module manages the decision-making algorithms and database. Its main element is a single-board DFRobot Lattepanda PC based on the Intel Atom Cherry Trail chip with 4 GB RAM and 64 GB internal memory. This module stores in a MySQL database the readings taken, which are used by the decision-making algorithms and in turn are arranged for synchronization and consultation with land-based communication stations. 

Regarding the communications infrastructure, BUSCAMOS-RobObs uses CAN bus, Ethernet network, WiFi and radio link. The CANopen fieldbus communicates all three nodes with the sbRIO master controller, enabling reliable and robust process data exchange between the controller core and the distributed periphery at a sufficiently high speed (500 kbps). The Ethernet network interconnects the sbRIO with the CPU, receiving the instructions and navigation parameters according to the results generated in real time by the decision-making algorithm. The vehicle communicates with the land-based station through WiFi and radio. It is equipped with omnidirectional WiFi antennas and high gain radio. The WiFi network, with its higher bandwidth, is designed to exchange large volumes of data. The low-bandwidth and long-range radio network guarantees communications during missions. For security reasons both networks have redundancy in critical tasks such as receiving instructions from the base, positioning and managing alerts.

### 3.4. Software Architecture

**Figure 4:**
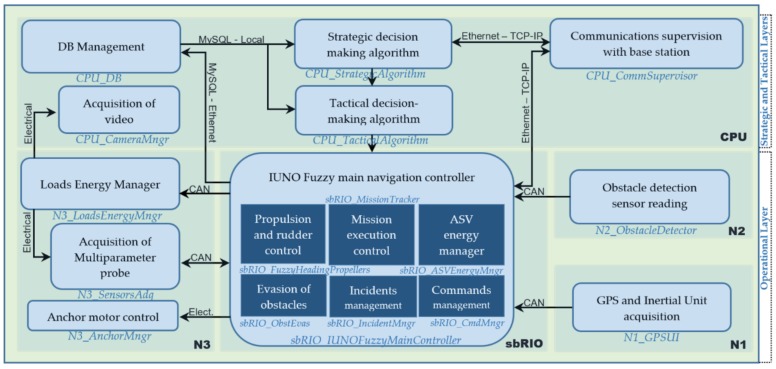
BUSCAMOS-RobObs software architecture.

The software module name is shown next to the name of the main code block algorithm implemented in each platform in Figure 4. The latter label is divided into two parts separated by a hyphen, where the first part indicates the hardware platform on which the software module is running (sbRIO, N1, N2, N3, CPU). The arrows indicate the direction of the data flow, and the label shows the type of communication or connection between the blocks. The layer to which each software element belongs is also displayed.

The hardware elements in the operational layer are basically micro-controllers and the sbRIO. The micro-controllers execute the functions shown in the figure periodically, triggered by time or event. For example, CAN readings every 10 ms, AD conversions every 5 ms, watchdog every 250 ms, RS232 acquisition every 250 ms. The algorithms/functions shown in the figure are the most important ones that each hardware element executes. In the case of software elements running on the CPU (belonging to the Strategic and Tactical layer) on the Windows 10 LTSB operating system, the software modules shown in the figure are programs running on this platform. This platform is powerful enough to run Fuzzy and real-time decision-making algorithms, as well as manage the MySQL database, make periodic communications checks and other lower-priority processes. A brief description of some of the software modules involved in the control is given in Table 2.

### 3.5. Command, Control, Communication and Computation Station: IUNO

**Figure 5:**
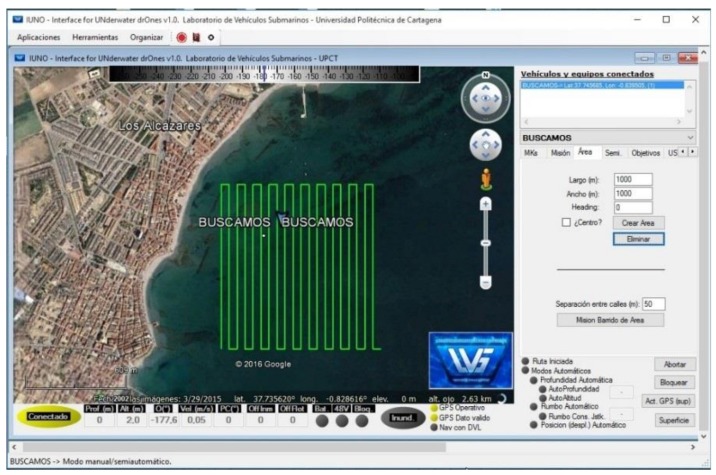
Graphical User Interface of IUNO software, showing exploration routes within a 1 km × 1 km box given by the four geographical coordinates (UTM) P1 (30T 690 4180), P2 (30T 690 4179), P7 (30T 691 4180) and P8 (30T 691 4179).

The “*Interface for Unmanned Drones*” (IUNO) is the base station control software, on which the human operators plan and send missions to the ASV. It was designed with the purpose of simplifying the management of unmanned vehicles by analysing the data collected by the vehicles and providing a fully automated vehicle management response to guarantee the success of the task. IUNO has a graphic user interface, showing the operator all the navigation information (vehicle location, power consumption and storage, historical tracks and programmed tracks, etc.). A screenshot of the IUNO software IUNO is shown in Figure 5.

IUNO has a wide range of functions, the most important being: multivehicle management, voice synthesizer, alarm event, geographical location and mission planner, fast navigation mode covering the planned geographical location given by coordinates (latitude and longitude), targets, and two navigation modes, manual and semiautomatic. The navigation modes are synchronized with the on-board sonar and GPS localizer and also incorporate OpenCPN nautical charts (.bsb, .kap, .map) to facilitate vehicle management. All the measured parameters are stored and sent to remote databases with a standard Keyhole Markup Language (KML) and Keyhole Markup Zip (KMZ) location marker, widely used for example in Google Earth. Being a command & control station, new missions can be launched by IUNO to any of the vehicles it is coordinating, while at the same time the human operator can cancel any of the missions assigned to any of the vehicles, whether it was previously assigned manually or automatically.

## 4. Design of Strategic Decision-Making Layer and Application to the Observation Mission in the Mar Menor

This section describes the design of the upper strategic decision-making layer, shown in Figure 2. At this level, a fuzzy algorithm processes the information from the vehicle’s sensors, its status and the environment to select the next exploration zone. For this, it has an up-to-date grid map of the entire exploration area, with a record of the status of each box, which depends on the time expired since the last exploration and the latest levels of physical and chemical parameters observed or recorded by other means. Among other parameters, the fuzzy system takes into account the vehicle’s power status to determine whether it has enough power to reach the next exploration box and when it will arrive, or whether it must anchor to recharge the batteries. To clarify this process, we added to the explanation of the algorithm an example of the exploration area, the Mar Menor, where all the tests and simulations were carried out. We will begin by describing this inland’s sea’s unique characteristics and the classification of the exploration zones.

### 4.1. Classification of Exploration Areas in the Mar Menor

**Figure 6:**
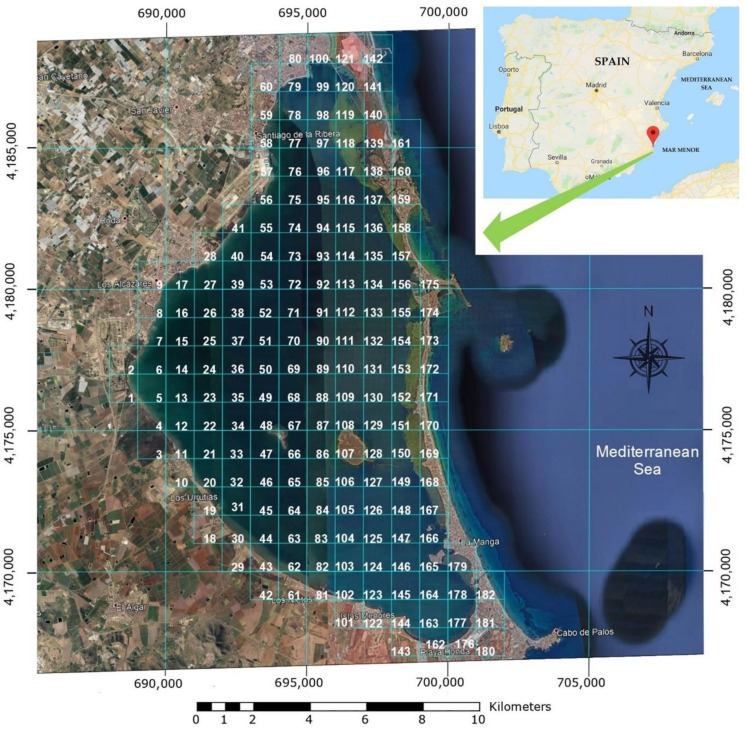
View of the Mar Menor divided into 182 boxes for exploration purposes and their GPS coordinates.

The Mar Menor, in the southeast of Spain (see Figure 6), is a unique and a valuable resource. Its uniqueness is due to its geographical characteristics (Europe’s largest salt lake, with an area of 180 km^2^), with a unique ecosystem in need of conservation. The Mar Menor is a valuable economic resource for the Region of Murcia for the tourism its attracts, for its beaches and good climate in [24] (mean maximum temperature 26 °C, mean minimum temperature 14 °C, 127 days of temperatures above 30°, and 3100 h of sun). This abundant supply of sunlight is an essential resource as an alternative energy source for the robotic mobile observatory.

Due to its unique characteristics, the Mar Menor has attracted the attention of numerous scientific researchers, such as biologists, environmental scientists, oceanographers, etc., especially since 2016, when the rising levels of chemical and biological contamination caused detectable changes in the composition and chemical balance of its waters. Algae proliferated due to the intense levels of human activities, discolouring the waters and affecting the ecosystem, since the clouded waters impeded the penetration of sunlight and this affected its flora and fauna. Tourism was also affected, since its beaches lost all of their previous 19 EU Blue Flags between 2016 and 2018. Blue Flags [25], awarded by the Foundation for Environmental Education (FEE), certify that a beach, marina, or sustainable touristic boat operation meets the Foundation’s environmental, educational, safety, and accessibility criteria.

The first step in effectively exploring the Mar Menor was to define a grid map, dividing the entire area into 180 1 km × 1 km boxes, using the National Topographic Map:Hoja 955 Torre-Pacheco, Hoja 956 San Javier and Hoja 978 Llano del Beal 1:50.000 scale (see Figure 6). The boxes were geo-referenced by GPS or UTM geographical coordinates stored in the on-board database. Data from previous exploration missions (historical archive) was also kept, including all the parameters measured by the on-board sensors or other information sources, like buoys or manual data collection. These parameters included environmental, physical, chemical and biological information (nitrate and phosphate contamination), turbidity, acidity, water density, oxygen, suspended particles, etc. All the parameter sets were tagged with their geographical location, date and time of acquisition.

### 4.2. Classification Strategy of Areas of Interest

**Figure 7:**
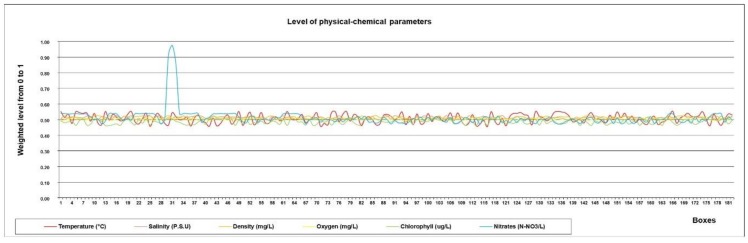
Normalized sensor values used for the simulation.

The mission’s strategic planning software layer used the information stored on the map to classify the state of each box. To do this, it has a fuzzy algorithm that whose input is the average stored values of the temperature, salinity, turbidity, oxygen, chlorophyll and nitrates variables in each one. On the basis of this information, the boxes were classified with the specific colour of a fuzzy state. To carry out the simulations, the values of environmental, physical, chemical and biological parameters of the 182 boxes were taken from a previous manual data collection in some representative areas and extrapolated to the other boxes, as shown in Figure 7.

**Figure 8:**
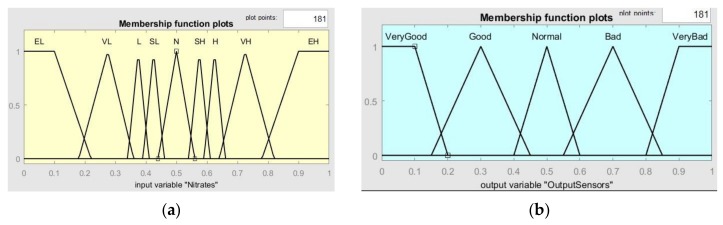
(**a**) Membership functions for each of the six sensors (shown for nitrates), where EL: extremely low, VL: very low, L: low, SL: slightly low, N: normal, SH: slightly high, H: high, VH: very high, EH: extremely high; (**b**) Output membership function for classifying the boxes according to sensor values.

The anomalous values of box 31 were manually added for simulation purposes only. The graph shows the values obtained from the 182 boxes, normalised and fitted to a scale from 0 to 1. The values are entered in the Fuzzy Sensor system to obtain the status of each box, as described below. The value for each sensor in each box is calculated as the mean of all the readings in that box (1 km^2^, in total the vessel travels 11 km) to avoid that a single measurement defines its value. Figure 8a depicts the fuzzy membership functions for the input sensor values (in this case, for nitrates values), and for the output value (Figure 8b). This output value defines the state of the grid, and therefore the colour of the area on the map. In total, 64 knowledge rules have been implemented in the fuzzy system, which in a summarized way follow the behaviour shown below:

- If the value of any of the inputs is EL or EH, then the output is VeryBad.
- If there are no EL or EH values but there is at least one VL or VH value, then the output is Bad.
- If there are no EL, EH, VL or VH values but there is at least one L or H value, then the output is Normal.
- If there are no EL, EH, VL, VH, L or H values but there is at least one LL or LH value, then the output is Good.
- If all input values are N, then the output is VeryGood.

**Figure 9:**
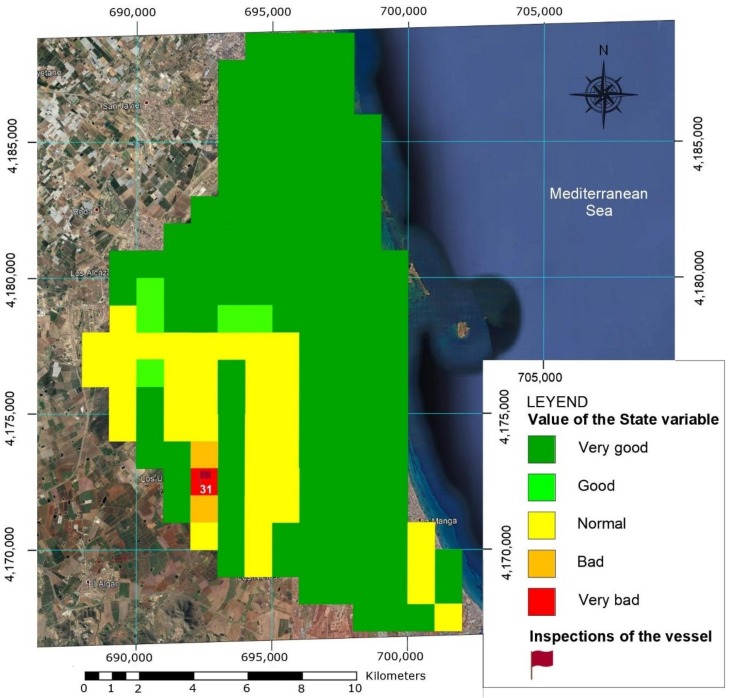
Colours of the state of each zone or box in the Mar Menor. The box number explored in the simulation is included.

Thus, for a specific experiment, each of the boxes on the map were coloured as shown in Figure 9: Very Good (green), Good (light green), Normal (yellow), Bad (orange) and Very Bad (red). A critical zone in a Very Bad state can be seen in Box 31, at the mouth of the Albujón river, where runoffs of agricultural nitrates reach the sea. Several Bad and Normal areas can also be seen, while the remainder is classified as Very Good.

### 4.3. Decisions Required to Navigate to the Area of Interest for Buoy-ASV Dual Operations

The decision on which box to explore is made by taking into account three fundamental parameters: its degree of interest (related to the state of the physical-chemical variables), the distance of the ASV to other squares, and the time expired since the last exploration. Equation (2) gives the rules used for the selection:

(2) $$\text{BoxInterest}=\frac{\left(\text{ObservationInterest}+\text{UpdateInterest}\right)}{\text{Distance}}$$

where:

$$\text{ObservationInterest}=\{\begin{array}{c} 24\left(\text{two years}\right)\;\text{if box State is Very Bad}\\ 12\;\left(\mathrm{one}\;\mathrm{year}\right)\;\text{if box State is Bad}\\ 0\;\text{otherwise} \end{array}\;\text{and UpdateInterest}=\text{time in months since last measurement}$$

The system calculates BoxInterest values for the whole map and selects the one with the highest value. Thus, from its initial starting point, the ASV will sail to and explore the indicated area of interest. Should its battery load fall below 20%, the vehicle will stop, drop its anchor and go into stand-by until the solar panels have recharged the batteries to 75%, after which it will wake up and continue the exploration. Should it receive an alert from an area, the State value of that area will become a priority (red) and the vessel will proceed to that area following the procedure outlined above.

During the journey to and exploration of the different zones, the values in the database are constantly updated with information on the 6 physical-chemical parameters and their date of capture.

### 4.4. Simulations to Validate the Navigation Process and Fuzzy Zone Classification Algorithm

**Figure 10:**
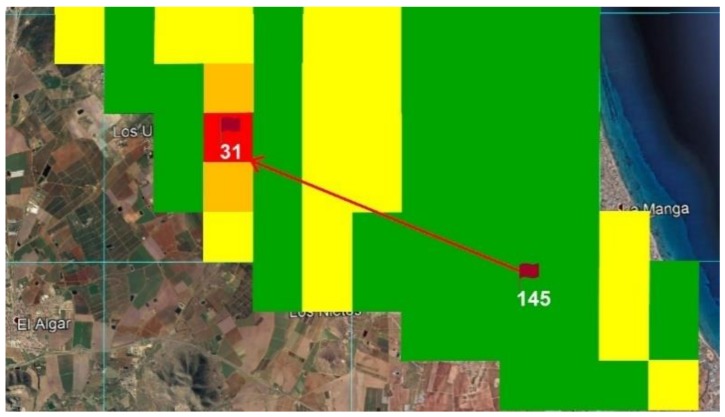
Map zone selected for the simulation with starting point at Box 145 and finishing point at Box 31.

This section describes an operational simulation performed on Simulink using the fuzzy classification algorithm and navigation strategy described above. A navigation step from Box 145 to Box 31 is simulated on the Mar Menor grid map, as seen in Figure 10.

**Figure 11:**
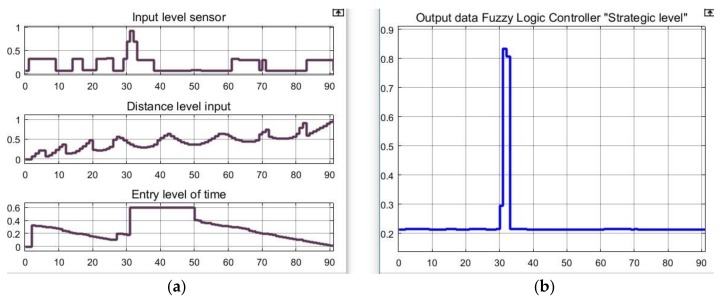
Graphics obtained from the Simulink model of the “Strategic level” control system. (**a**) Input values of sensors, distance and time; (**b**) Output value (box 31 selected).

**Figure 12:**
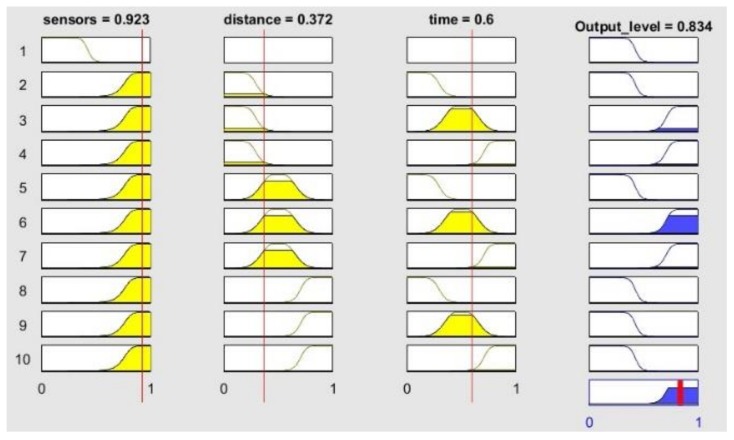
Fuzzy rules for the simulated case.

The simulation sequence was as follows: while in Box 145 BUSCAMOS-RobObs receives an alert from Box 31, where a high nitrates reading has been detected in the fuzzy map output shown in Figure 10. New *BoxInterest* values are then computed for the boxes (Figure 11b shows only the first 90 boxes). These values are calculated from the three parameters shown in Figure 11a and Equation (2): the first scope shows the values obtained to establish the colour intensities on the map in Figure 9; the second scope shows the values of the distance from the ASV’s current position to the other boxes; while the third shows the time that has passed since each box was explored. Figure 12 gives the rule set that generates the fuzzy output from the input values. As can be seen, a total of 10 rules were defined to model the box selection procedure.

### 4.5. Energy Management Simulations

As the ASV must travel by means of the energy captured by its solar modules, we must know the coordinates of the target box, the time of year, hours of solar radiation received, the power of the vessel’s photovoltaic generator, the generator’s efficiency, and any losses in the system.

**Figure 13:**
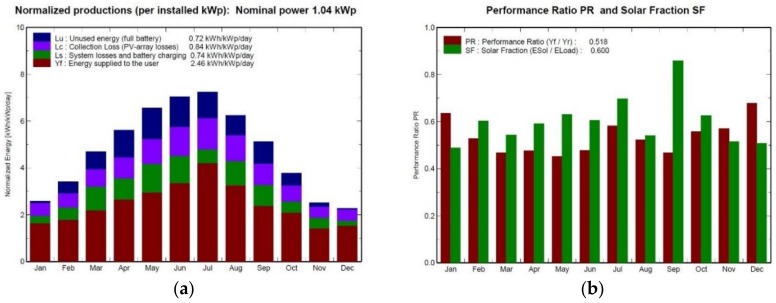
Results of PVSYST simulations: energy generation (**a**) and performance ratio (PR); (**b**) for each month of the year.

**Figure 14:**
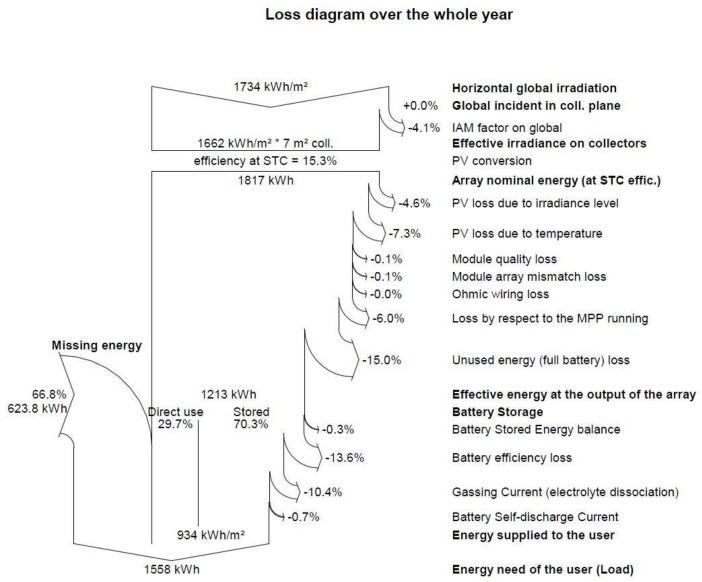
Energy losses of the PV system, balance between final photovoltaic energy generation and total demand, showing the requirement for a back-up generating system. (Performed with PVSYST V5.05).

The energy management system is described in Section 3.1. The energy balance between the generating system (maximum 1040 Wp), battery storage and energy consumption is shown in Figure 13 and Figure 14. The photovoltaic energy generated in any month is clearly not enough to cover the power demand, since there are losses in the wiring and electrical systems and batteries, due to temperature changes and the efficiency of the electronic equipment, horizontal panels, etc. The simulation results after considering all these factors are shown in Figure 13, which shows that the power required to supply the system all year round would be 623.8 kWh. Since the system had to be completely autonomous and we only had the battery storage to fall back on, the vehicle was designed as a dual system.

The environmental data (irradiation, daily average temperature) are provided by METEONORM V6.1.0.23, which is a complete database; the data were obtained for San Javier, Murcia, Spain (lat. 37.8 N, long. 0.8 W). The results of the simulations are shown in Table 3 and Table 4.

Since the fully charged batteries can operate for 6 h, the daily exploration of each box was divided into time bands (see Table 4), one in the morning, another at noon and the last one in the late afternoon. When recharging batteries and at night, the vehicle remains anchored to the seabed, in stand-by mode.

This sampling strategy was operated through different levels of interconnected fuzzy control techniques to achieve a robust strategic control system and effectively increase the autonomy of the proposed robotic observatory.

## 5. Experimental Tests and Results

This section describes in detail a 10-day mission to test the vessel’s power system, autonomy and long-term operations. The test began on 20 July 2018 at 8:00 in the morning. Prior to starting the experiment, the different systems were checked as follows: battery power, photovoltaic recharge, manual test of propellers, rudder and anchor deployment, control system auto-diagnosis module by means of the IUNO remote monitoring system, verification of sensors and devices (GPS, multiparameter probe, scanning sonar, etc.), and communications between the vessel and base station.

**Figure 15:**
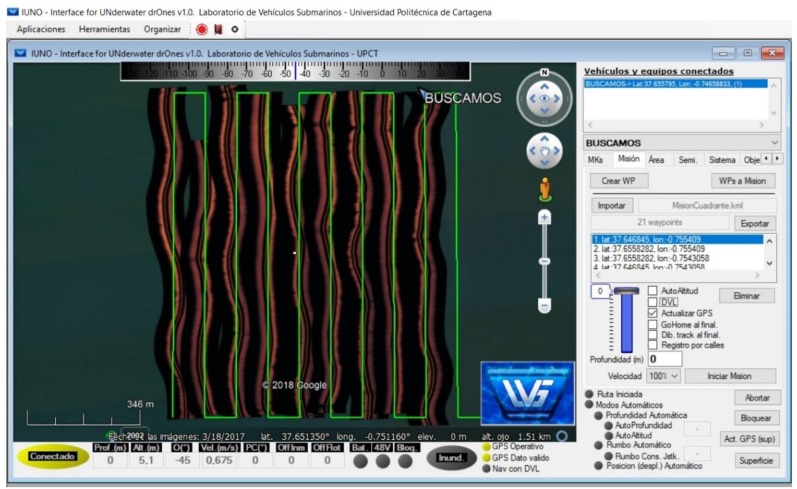
Sonar image of the actual route of the vehicle in Box 102 superimposed on the route setpoint trace. Representation in HMI of IUNO.

**Figure 16:**
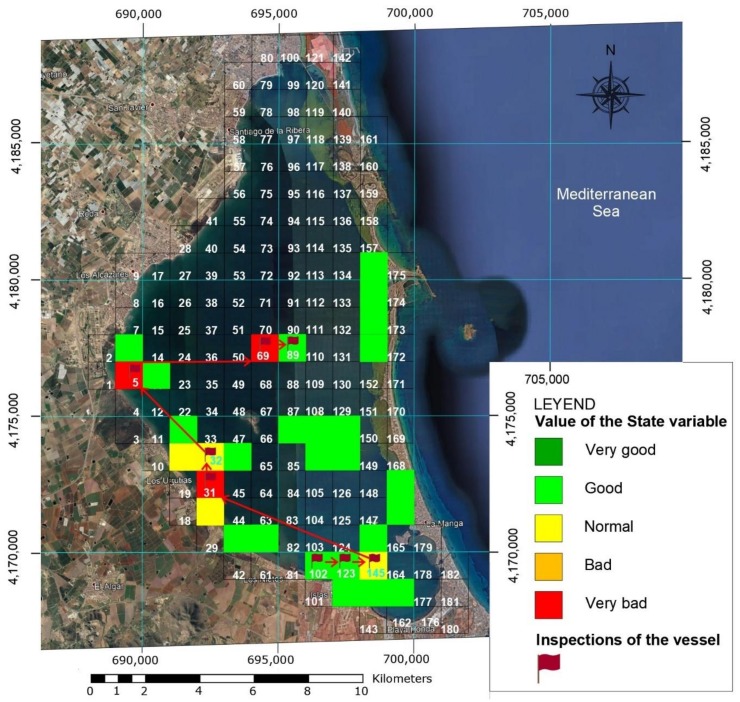
Box colour scheme. Boxes with a number and colour are the ones explored and therefore have updated data. The coloured squares without numbers are previously collected values. Uncoloured boxes are those for which no data are available and require monitoring. The values in red (alarms) simulate possible spills or contamination.

The vessel was launched with fully charged batteries in Box 102, near the *Los Nietos* Yacht Club (see Figure 6). The database for the decision making algorithms was loaded with previously acquired data from the exploration boxes. The status colours of the boxes and the mission results can be seen in Figure 16. The first box inspected was N° 102, (UTM coordinates: X: 696,000, Y: 4,170,000). The exploration route followed parallel lines separated by 100 m over the entire length of the grid. During the tour, sonar images, videos of the seabed and water parameters were recorded (see Figure 15). The inspection of Box 102 ended at 11:00 a.m., with a power consumption of 2.5 kWh. The values obtained by the multiparameter probe are shown in Table 5. These data were stored in the database together with the corresponding timestamp, which can influence the decision on the next box to visit.

Having only updated data of a few nearby areas (green boxes around 102 in Figure 16), the criterion of greatest weight in the decision making was the proximity of contiguous zones. We thus proceeded to inspect in the same way and with the same type of sampling Boxes 123 (UTM coordinates: X: 697,000, Y: 4,170,000) and 145 (UTM coordinates: X: 698,000; Y: 4,170,000), see red flags in Figure 16). Box 123 was completed at 16:00 h, with an energy consumption of 3.8 kWh. The increased power consumption was mostly due to changing course and a strong wind.

Having completed 52% of the inspection of Box 145 at 19:00, a nitrates alert in Box 31 was simulated from the IUNO system in the base station. This susceptible area is at the mouth of a river and should in fact be permanently monitored by a fixed buoy, as it is a known critical dumping area. Before this event, the vessel was on its way to Box 31, as the decision-making algorithms had selected this for monitoring next. After travelling 4 km and consuming 4.7 kWh, the vessel stopped as the battery charge was below 20%. The algorithms decided that the best strategy was to switch to recharge-only mode and anchor overnight at 1.85 km from Box 31.

The vessel remained anchored to recharge above 75% (8917 Wh) until 3:00 p.m. on 23 July and then continued to Box 31, using 1.0 kWh. The exploration of Box 31 began at 16.00 h and ended at 20:00 h, after which it began to explore Box 32, where at 9:00 p.m. it dropped anchor for an overnight stay (see Figure 16).

At 7:00 a.m. on July 24, the exploration of Box 32 resumed but stopped again at 11:00 a.m., when another simulated alert was received from Box 5 (UTM coordinates: X: 690,000; Y: 4,176,000). The ASV the travelled 1 km to that box until the batteries reached 20% and entered recharge mode at UTM coordinates X: 691,284; Y: 4,174,708, where it stayed until 26 July, when the charge rose above 75%. It then went 1.83 km to Box 5, and began an inspection but dropped anchor at 21:00 h. At 7:00 a.m. on 27 July, the ASV resumed the exploration of Box 5, which ended at 8:00 a.m., when it received another alert from Box 69, proceeding to that box. After 2 km the battery level again fell below 20%, when it again entered recharge mode at UTM coordinates: X: 692,000; Y: 4,177,000. It remained here until two days later, 29 July, with the batteries over 75% and went to Box 69, beginning the exploration at 16:00 h, ending at 9:00 p.m. and going into standby. At 7:00 a.m. on 30 July it began exploring Box 89 until the end of the test at 12:00 h, having been working autonomously for 10 days. In Figure 16 shows the course followed. The target boxes are marked with red flags.

**Figure 17:**
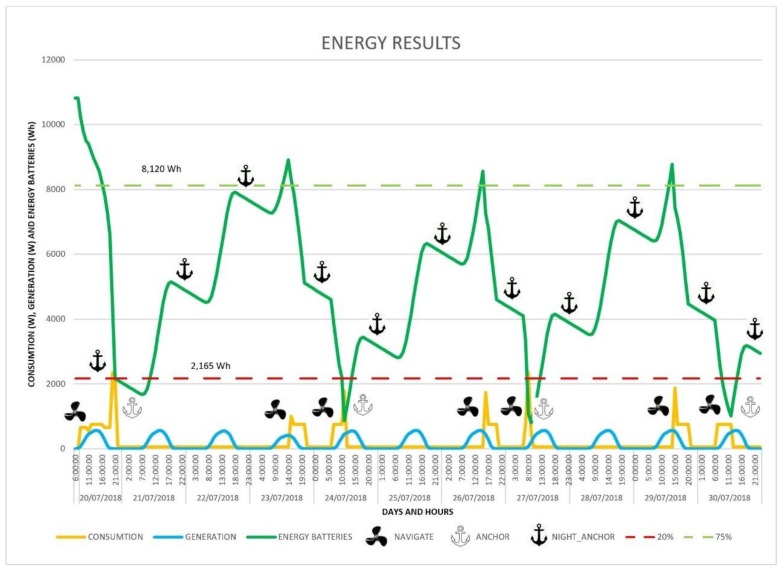
Energy generation and consumption during the 10-day autonomous navigation experiment. ‘Y’ axis has the same scale for all the curves, but the units are different: Watt for orange and blue curves, Watt-hour for green curve.

The final result of the energy balance can be seen in the graph in Figure 17, which in addition to the ASV consumption (orange curve, in W) shows the power generated by the photovoltaic system (blue curve, in W), the energy stored in the batteries (green curve, in Wh), the times when the ASV sets sail, and when the anchor is dropped to recharge overnight. Note that though the units are different, the scale is the same for the curves. The white anchor symbol represents a stop to recharge batteries, a black anchor means an overnight stop, and the propeller shows the time when the boat gets under way. The horizontal dashed lines represent the minimum battery charge to anchor the vessel (in red, 20%), and the minimum charge to get under way (in green, 75%).

## 6. Conclusions and Future Works

This article has described the novel BUSCAMOS-RobObs dual robot-buoy mobile observatory, which was specifically designed to maintain a permanent presence in shallow waters, by combining the main advantages of autonomous vehicles and buoys to overcome their limitations and challenges. Although many solutions have been proposed in the literature for monitoring marine environments, as far as we know none of them satisfies the power requirements for long-term explorations.

Since an autonomous power system is fundamental in achieving a permanent presence, the article gives an in-depth analysis of the system’s unique power management system, including its design (1040 Wp photovoltaic generator, 8 solar panels and 10,740 Wh batteries), a simulation of its seasonal performance (summer battery recharging is 50% faster than winter). Actual data on BUSCAMOS-RobObs energy consumption and energy management was used in an experimental 10-day mission in July 2018 in Europe’s largest inland sea, the Mar Menor, with 186 Km^2^ in the southeast of Spain.

BUSCAMOS-RobObs is a dual system because it combines the advantages of ASV mobility with the stability of a fixed buoy. As an autonomous vehicle it can travel to selected points to measure water quality and scan the seabed. When acting as a buoy it can anchor over a point either to take continuous measurements or recharge its batteries. The ability to recharge batteries when anchored to the seabed not only means faster recharging, since the position control systems are on standby, but also ensures that the vehicle will not drift due to currents or winds. This ability is what gives the system an enormous autonomy to carry out long-term missions.

As we concluded from the review of the state-of-the-art, BUSCAMOS-RobObs provides a novel solution and achieves a permanent autonomous presence in lakes or shallow coastal waters. This improves the autonomy in monitoring water quality parameters and avoids the problems associated with the deployment of a large number of fixed marine observatories (buoys), which influence and impact maritime traffic, the environment, tourism and the cost involved.

Another notable contribution, in line with the objective of minimising the vehicle’s energy consumption, is that its fuzzy mission management system takes into account the status of the vehicle’s batteries and the distance to alternative exploration areas to select the next mission zone. Also described was the fuzzy system that uses the measurements of the vehicle’s sensors to characterise the water quality and possible contamination to generate a colour map of the status of the area, as described in Section 5. This map is updated as new measures are taken, and is also considered when determining the next area to explore.

The viability of the approach was demonstrated by a real 10-day monitoring mission carried out in the Mar Menor, and by several simulations of the vehicle’s performance and power consumption. The results of both the simulations and the experimental mission were found to be satisfactory, reaching the objectives of maintaining a permanent monitoring presence for a period of 10 days and travelling 92.28 Kms without human intervention. The data collected were monitored and stored remotely in the IUNO system. The energy management system also performed well, being able to navigate for the entire period, anchoring each night and selecting the 4 stops for recharging (see Figure 17).

No limitations were observed in the operation, apart from the one derived from the scope of BUSCAMOS-RobObs application. Since the anchoring depth limit is 8 m, it cannot operate in deeper waters, which is why we specify that it is a solution for permanent monitoring of shallow waters. It is also necessary to take into account the prevailing weather conditions (especially wind), wave heights and tides in the area, which may hamper operations even with recharged batteries. Still, it is a solution that can be applied to permanently monitor a multitude of lagoons and coastal seas around the world.

Given the large area to be covered in monitoring the Mar Menor (186 Km^2^) and the speed of the vessel, we estimate that it would take up to 1 year to complete a full autonomous exploration. In the future, deploying more vehicles could reduce this time. The number of vehicles depends on the maximum allowed age for the data gathered and the available budget. For example, we estimate that 3 vehicles could cover the whole area in 3–4 months. However, it should be noted that this is a monitoring vehicle and is not equipped to intervene, and therefore it is acceptable that it takes a while for the vessel to reach a box in which an alarm has raised. Should a spill happen, other means must be deployed to deal with it. 

Regarding future work, we are currently planning to: (i) perform longer monitoring missions to improve the efficiency of the solar capture of the photovoltaic system and improve the capacity of the batteries; (ii) carry out missions in autumn and spring with fewer hours of daylight and more cloud cover in less favourable areas; (iii) manage recharge periods by means of new algorithms so as to start the vehicle’s activity with lower power levels; (iv) introduce new sources of renewable energy, such as wind turbines, and (v) automate post-processing of data using big data techniques.

## Figures and Tables

**Table 1:** Parameters describing the PV modules and batteries.

PV Modules: Enecom Italia, HF130		Batteries: Lithium-Manganese, Torqeedo, Power 26–104	
Solar power	130 (Wp)	Voltage	25.9 (V)
V_oc_	20.01 (V)	Nominal load	104 (Ah)
V_mpp_	16.86 (V)	Total units	4 batteries (2 series/2 parallel)
I_sc_	8.61 (A)	**Total energy stored**	**10,740 kWh**
I_mpp_	8.10 (A)		
Total units	8 modules (4 series/2 parallel)		
**Total power**	**1040 Wp**		

**Table 2:** Description of software modules.

- **N1_GPSUI**: obtains and validates GPS position and course of the inertial unit - **N2_ObstacleDetector**: detects obstacles and sends alerts to sbRIO - **N3_LoadsEnergyMngr**: manages expendable loads and energy modes (buoy, recharge, ASV) - **N3_SensorAdq**: acquires and supervises the multi-parametric probe and sonars - **N3_AnchorMngr**: manages the anchor depending on the active energetic mode	- **CPU_DB**: manages the connection with the MySQL database and the data stored on it (geo-referenced navigation and sensor data), which is then used by the decision-making algorithms - **CPU_CommSupervisor**: manages communication link with the base station - **CPU_CameraMngr**: manages the on-board cameras
- **sbRIO_IUNOFuzzyMainController**: set of software implementing the previously described operational level through the following functions: ○ **sbRIO_FuzzyHeadingPropellers**: implements fuzzy algorithms to correct the ASV’s heading and speed, as well as an auto-adaptable K factor for PIDs **sbRIO_MissionTracker**: supervises the route of the mission. Together with the former, they both constitute the fuzzy navigation controller **sbRIO_ASVEnergyMngr**: energy management controller **sbRIO_ObstEvas**: generates a new path when an obstacle is detected by node N2. **sbRIO_IncidentMngr**: sends incidents to the base station (errors and alerts), depending on their severity. Manages self-restoration of some solvable incidents **sbRIO_CmdMngr**: manages the connection and interface with the base station, sending and receiving commands	

**Table 3:** Summary of the results of the simulation obtained with PVSYS.

Annual Recharges	Energy for Recharging	Annual Power Requirements	Useful Solar Power	Energy Gap	Performance Ratio (PR)
143	10.816 kWh	1558 kWh	934 kWh/year	624 kWh	51.8%

**Table 4:** Box exploration time bands, stoppages and recharging periods in different seasons of the year.

Season	Daily Exploration Periods	Recharging Periods	Night Anchorage Periods
Summer	07:00–08:00 12:00–14:00 18:00–20:00	08:00–12:00 14:00–18:00 20:00–21:00	21:00–07:00
Spring	08:00–09:00 12:00–14:00	09:00–12:00 14:00–19:00	19:00–08:00
Autumn	07:30–09:30 12:00–14:00	09:30–12:00 14:00–19:30	19:30–07:30
Winter	08:30–09:30 12:00–14:00	09:30–12:00 14:00–17:00	17:00–08:30

**Table 5:** Average parameter values in Box 102.

Data	Average Value
Temperature	21.75 °C
Salinity	42.25 P.S.U
Density	0.12 mg/L
Oxygen	6.84 mg/L
Chlorophyll	2.28 µg/L
Nitrates	0.98 mg N-NO3-/L
